# Multimodal signals: ultraviolet reflectance and chemical cues in stomatopod agonistic encounters

**DOI:** 10.1098/rsos.160329

**Published:** 2016-08-03

**Authors:** Amanda M. Franklin, N. Justin Marshall, Sara M. Lewis

**Affiliations:** 1 Department of Biology, Tufts University, Medford, MA 02155, USA; 2 Sensory Neurobiology Group, Queensland Brain Institute, University of Queensland, Brisbane, Queensland 4072, Australia

**Keywords:** multimodal signal, complex signal, agonistic, stomatopod, crustacean, communication

## Abstract

Complex signals are commonly used during intraspecific contests over resources to assess an opponent's fighting ability and/or aggressive state. Stomatopod crustaceans may use complex signals when competing aggressively for refuges. Before physical attacks, stomatopods assess their opponents using chemical cues and perform threat displays showing a coloured patch, the meral spot. In some species, this spot reflects UV. However, despite their complex visual system with up to 20 photoreceptor classes, we do not know if stomatopods use chromatic or achromatic signals in contests. In a field study, we found that *Neogonodactylus oerstedii* meral spot luminance varies with sex, habitat and, more weakly, body length. Next, we conducted an experimental manipulation which demonstrated that both chemical cues and chromatic signals are used during contests. In the absence of chemical cues, stomatopods approached an occupied refuge more quickly and performed offensive behaviours at a lower rate. When UV reflectance was absent, stomatopods performed offensive behaviours more frequently and contest duration trended towards shorter fights. These results provide new evidence that UV reflectance and/or visible spectrum luminance is used to amplify threat displays. Our results are the first to demonstrate that chemical and chromatic cues comprise a multimodal signal in stomatopod contests.

## 1. Introduction

In many animal interactions, complex signals are used to improve communication ability. A complex signal is when more than one signal is assessed simultaneously or sequentially within a given interaction (see [1] for a review). If such signals use more than one modality (e.g. visual, acoustic and chemical), then they are multimodal signals, otherwise they are known as unimodal signals. In these interactions, each signal may transmit different information. For example, the black cap of the American goldfinch, *Carduelis tristis*, provides information about social interactions whilst the plumage and bill coloration provide information about infection [2]. Using distinct signals to transmit different information can reduce the response time of the receiver if the receiver processes each signal simultaneously or if the signaller uses one signal to alert the receiver to the second signal [1]. Alternatively, different signals may convey redundant information. This could be beneficial if the receiver responds more appropriately to two signals than to one [3], or if particular signals are more effective under different environmental conditions [4]. Presumably due to these benefits, complex signals are common in many animal interactions, including mate choice [5–7], intraspecific competition [8–10] and even predator/prey interactions [11,12].

Many animals rely on complex signals to minimize the risk of injury during intraspecific fights over resources (e.g. mates, food or territories). In general, animals use signals in these situations to assess their opponent's fighting ability or aggressive state [4,8,13,14]. This allows individuals to avoid engaging in fights that they are unlikely to win. One use of complex signals in these interactions is to increase the accuracy of the receiver response [1]. For example, in swamp sparrow contests the combination of soft song and wing waving by territorial males more accurately predicts an attack on conspecifics than each display in isolation [8]. Complex signals may also be used to convey information about different aspects of the signaller. Eland antelope males use knee-clicking to signal their body size, whereas dewlap size signals age (and presumably fighting experience [13]). Thus, the interaction between two signals may facilitate more accurate assessment of opponents during agonistic encounters.

While research into complex signals is rapidly expanding, we still know surprisingly little about signals used by certain taxa. Stomatopods are excellent subjects for studying complex signals in agonistic encounters due to their highly territorial behaviour, use of chemical cues and their complex vision. Also known as mantis shrimp, these tropical crustaceans will fight aggressively with conspecifics over possession of a refuge (a suitably sized hole in coral or rock). During fights, they perform a display known as the meral spread in which the second maxillipeds are pulled laterally, displaying the meral spots. These spots vary in colour between species, but may have an ultraviolet (UV) component [15].

It is likely that stomatopods use the UV reflectance of the meral spot as a signal in agonistic encounters. Stomatopods have up to 20 photoreceptor classes [16] with five photoreceptor classes in the UV [17,18]. Although their colour discrimination may be coarse [19], the disproportionate number of UV photoreceptors suggests that UV vision is important in stomatopods. Currently, we know little about stomatopod chromatic and/or achromatic signalling, except that it is likely to be used in mate choice in *Haptosquilla trispinosa* [20]. In this case, the manipulations conducted by Chiou *et al*. [20] simultaneously altered hue, luminance and polarization so it is unclear which component (or combination) is used as a signal.

Stomatopods' use of chemical cues has been more thoroughly studied. It has been demonstrated that stomatopods use chemical cues in agonistic encounters to detect their opponent's size [21] and whether or not they have recently fought that particular opponent [22,23]. Thus, it is plausible that stomatopods use both chromatic signals and chemical cues in agonistic encounters.

The stomatopod *Neogonodactylus oerstedii* [24] is an ideal species in which to study UV signalling. We know they possess five photoreceptor classes in the UV [17,18] and have a purple (to our vision) meral spot with UV reflectance, as shown here. They are found throughout the Caribbean in shallow waters, where they are illuminated by broad band sunlight including 300–700 nm [25]. *Neogonodactlyus oerstedii* reside in cavities with circular openings in coral rubble or rock. These refuges are important for avoiding predators, processing food, mating and brooding eggs [21]. Thus, stomatopods will fight each other aggressively over ownership of these refuges. Contests are generally won by the resident stomatopod or the larger of two stomatopods [21].

Here, we conduct two studies to investigate the signalling role of the meral spot. The first was a field study to investigate whether intraspecific variation in meral spot hue and luminance could signal stomatopod body size and, thus, fighting ability. The second study was an experimental manipulation to determine whether UV reflectance of the meral spot operates as an aggressive signal in agonistic encounters, and to investigate how UV reflectance and chemical cues may act as a multimodal signal in agonistic encounters. Specifically, we tested whether UV reflectance and chemical cues convey redundant information or different information to the receiver.

## 2. Material and methods

### 2.1. Animal collection and husbandry

This study was conducted at the Smithsonian Institution's field station at Carrie Bow Cay, Belize (16°48′ 9^″^ N, −88°4′ 55^″^ W). *Neogonodactylus oerstedii* were collected from coral rubble, rocks and discarded conch shells (hereafter called ‘rubble’) while snorkelling in shallow (less than 2 m) mixed seagrass (*Thalassia testudinum* and *Syringodium filiforme*) beds and consolidated coral rubble. Stomatopods were extracted from rubble using a pick, and their body length (tip of rostrum to tip of telson), wet weight and sex were recorded. We housed stomatopods individually in 19 l, white plastic buckets with running seawater. Each stomatopod was provided with a refuge consisting of a 25 ml falcon tube wrapped in black duct tape with the tapered end removed (15 × 105 mm). To mimic natural conditions, large stomatopods (more than 40 mm) received a refuge with a 10 mm opening, and small stomatopods (less than 40 mm) a 7 mm opening.

### 2.2. Field study: variation in meral spot reflectance

To investigate variation in meral spot reflectance, we collected 16 females and 13 males and recorded their habitat of collection, body length and body mass. Stomatopods were collected from within adjacent seagrass or rubble habitats; only stomatopods more than 1 m from the habitat boundary were included in this study.

**Figure 1.**
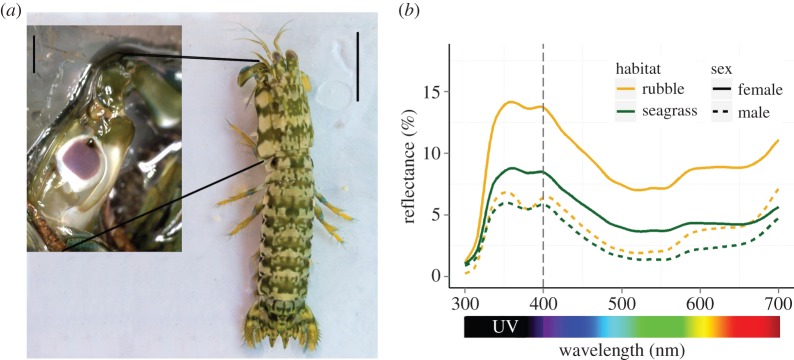
Meral spot of *Neogonodactylus oerstedii*. (*a*) Dorsal view of a male (scale indicates 10 mm). Inset shows the ‘purple’ meral spot on the inside of the raptorial appendage (scale indicates 2 mm). (*b*) Mean spectral reflectance of ‘purple’ area of male and female meral spots collected from two habitats in Belize. Grey dashed line indicates separation between the UV and visible spectra used in luminance analysis (area under the curve). Sample sizes are as follows: female, rubble: *n* = 10; female, seagrass: *n* = 6; male, rubble: *n* = 4; male, seagrass: *n* = 9.

We recorded the spectrum of the central coloured part of the meral spot (figure 1*a*) in the laboratory using a JAZ spectrophotometer (Ocean Optics, Dunedin, USA) with a PX-2 pulsed xenon light source. The reflectance was recorded between 300 and 700 nm (UV: 300–400 nm; visible spectrum: 400–700 nm) and measured relative to a WS-1 white standard. To mimic natural conditions, the light source was set at 45° to the meral spot surface and the collector was perpendicular [26,27]. Light source and collecting probes were 600 µm UV-VIS fibre-optic cables (Ocean Optics, Dunedin, USA) with collimating lenses attached to the end. Both were fixed in position to ensure a standard distance between probes and sample. Live stomatopods were immobilized by cooling them in a freezer (−13°C) for 30 min in 25 ml of water, and then pinning them out in a Petri dish with the meral spot facing up. The Petri dish was filled with just enough seawater to cover the meral spot [27]. All reflectance measurements were recorded in a dark box with only the light source illuminating the sample. We recorded two measurements of both the left and right meral spots.

### 2.3. Experimental manipulation: effect of UV reflectance and chemical cues on agonistic behaviours

#### 2.3.1. Manipulation of chemical and UV signals

We used a fully crossed, two factor design to investigate the effects of UV reflectance and chemical cues on intruder behaviour during agonistic encounters. In these behavioural trials, residents were given a refuge whereas intruders were not. Stomatopods were assigned to same-sex pairs based on similar body lengths (within 6% [21]). One member of each pair was randomly assigned to be the resident and the other was the intruder.

Pairs were then randomly allocated into one of four treatments that manipulated what information was available to the intruder: residents either had meral spot UV reflectance present (designated as UV+) or absent (UV−), and intruders could either detect chemical cues (CC+) or they could not (CC−). This resulted in four treatment groups: UV+/CC+ (control), UV+/CC− (effect of chemical cues), UV−/CC+ (effect of UV reflectance), UV−/CC− (combined effect of UV reflectance and chemical cues; electronic supplementary material, figure S1). Ten stomatopod pairs were tested in each group.

To manipulate intruders' ability to detect chemical cues, stomatopods were anaesthetized by cooling. Intruders were placed in a 50 ml container with 25 ml of seawater and cooled in a freezer (−13°C) for 20 min. CC− intruders had their antennae dipped in freshwater for 60 s, which has been demonstrated to temporarily remove their ability to sense chemical cues [28]. After treatment, stomatopods were warmed to ambient water temperature over 20 min and then allowed to recover overnight in their housing bucket. CC+ intruders were treated similarly except their antennae were dipped in seawater as a control.

**Figure 2.**
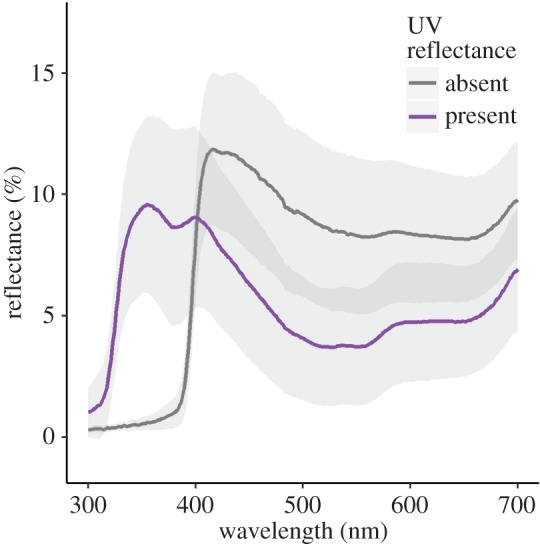
The effect of paint treatment on meral spot reflectance (mean ± s.d.) in *Neogonodactylus oerstedii*. Painted stomatopods (grey line) had reduced UV reflectance (300–400 nm) and increased reflectance in the visible and near infrared (400–800 nm) wavelengths compared with controls (purple line), to maintain similar overall luminance between treatments.

To manipulate the UV reflectance of meral spots, the resident in each pair was anaesthetized by cooling in the freezer (−13°C) for 30 min (this treatment required more time to complete than the intruder treatment). Each UV− stomatopod was pinned in a small Petri dish to expose the meral spot. The spot was dried with a cotton bud then painted with sunscreen (Banana Boat Sport SPF 50, USA) and allowed to dry for 45 s. A 1 : 1 mixture of superglue (Krazy Glue Elmer Products, USA) and clear nail varnish (Milani Cosmetics, USA) was painted over the sunscreen to seal it and increase luminance (area under the spectral curve) in the visible spectrum (figure 2). We increased luminance to ensure that overall luminance was similar for UV+ and UV− individuals. This mix was allowed to dry for 90 s. The same procedure was then performed on the other meral spot. After treatment, stomatopods were warmed to ambient water temperature over 20 min and then allowed to recover overnight in their housing bucket. UV+ stomatopods experienced the same cooling and recovery conditions, but were not painted. No paint could be found that retained spectral properties or only brightened the meral spot.

To investigate whether the scent of the paint mixture altered stomatopod behaviour, we performed a choice experiment at Tufts University with *N. oerstedii* obtained commercially (KB Marine Life, FL, USA). Sixteen stomatopods were allowed to choose between two refuges, one painted (as above) on the inside and the other untreated. We detected no preference for a refuge with or without paint treatment; nine out of 16 stomatopods choose the painted refuge (*p*  =  0.17). Thus, any behavioural changes detected are likely due to the visual aspect of the treatment, not a chemical aspect.

**Figure 3.**
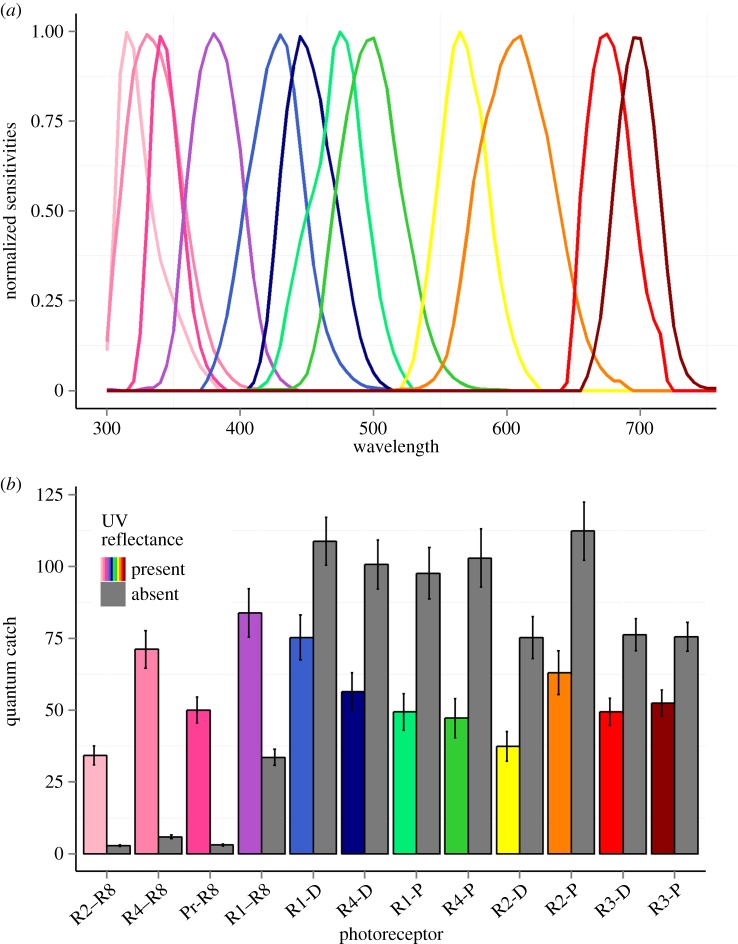
*Neogonodactylus oerstedii* spectral sensitivities and quantum catch. (*a*) The spectral sensitivities of *N. oerstedii*'s 12 photoreceptors involved in UV and visible spectrum light detection. (*b*) The quantum catch of each photoreceptor for a meral spot with UV present (coloured bars) or absent (grey bars). Quantum catch was calculated by multiplying a photoreceptor's spectral sensitivity by the reflectance spectrum of the meral spot and summing the result for that photoreceptor. Photoreceptors are labelled according to their row number in the midband (R1–R4) or ‘Pr’ if located in the hemispheres and whether proximal tier (P), distal tier (D) or R8 cell. Colours in each panel correspond to the same photoreceptor. Bars are mean ± s.e.m.

To assess how stomatopods might perceive our experimental manipulation, we conducted a basic visual analysis to estimate ‘quantum catch’, the amount of photons detected by each photoreceptor type [29]. After behavioural experiments, we recorded two spectral measurements from either the left or right meral spot from 19 UV+ and 15 UV− stomatopods. The two spectra were averaged and then multiplied by the known spectral sensitivity for each of *N. oerstedii*'s 12 photoreceptors that are associated with chromatic vision in the UV and visible spectrum [18,30] (figure 3*a*). The result was summed for each photoreceptor and then averaged for each treatment group.

#### 2.3.2. Measuring agonistic behaviours

Behavioural trials were performed at the shoreline between 09.00 and 15.00 to ensure that lighting was as natural as possible. Trials were conducted in a white plastic tub (60 × 36 × 12 cm) with a 3 cm deep layer of sand and 5 cm depth of seawater (approx. 15 l). Each tub was divided in half with a removable opaque plastic divider. The resident stomatopod was placed at one end of the tub, in its refuge from the housing bucket, and then allowed 30 min acclimation time. After 15 min of acclimation time, 10 l of fresh seawater was siphoned into the tub in case chemical cues from the resident accumulate. After this, the intruder was placed at the centre of the second compartment, without its refuge and received 10 min acclimation. At this time, the opaque barrier was removed and recording of behaviours began. All trials were recorded from above with a GoPro video camera (Hero 3+ Black edition, USA; settings: 60 f.p.s., 1080p, medium f.o.v.) and scored blind. Intruder behaviours recorded from the video footage included: latency until approached refuge, speed of approach, closest proximity to burrow, latency until first offensive behaviour, duration of fight, number of antennal flicks (rapid, lateral back and forth movement of antennule [31]), number of offensive behaviours, number of defensive behaviours and the winner of the fight. Offensive behaviours included ‘strike’, a blow delivered by one or both of the enlarged second maxillipeds; ‘lunge’, a short, rapid forward movement towards opponent; and ‘meral spread’, an outward spreading of raptorial second maxillipeds. We recorded one defensive behaviour, ‘coil’, where the stomatopod curls up so that its head is above the telson (see the electronic supplementary material, video S1 and [31] for further description and visuals of behaviours). Residents tended to remain in their refuges and were not visible from above, so we did not record their behaviours. Recording of behaviours concluded as soon as a clear winner was observed. If there was no interaction or no clear winner, the trial was ended after 30 min. At this time, stomatopods were returned to their housing buckets and the experiment was set up with fresh seawater for the next trial.

### 2.4. Statistical analysis

All statistical analyses were conducted in R v. 3.1.1 [32] and statistical model details are indicated in the electronic supplementary material, table S1.

#### 2.4.1. Field study

For the field study, we used generalized linear models (GLMs; R: glm) to assess whether meral spot hue or luminance correlated with sex (male/female), habitat (seagrass/rubble), body length or body condition. Spectra were compiled using CLR [33], and hue (peak wavelength) and luminance (area under the curve) were calculated using RCLR [34]. Luminance was calculated separately for the visible spectrum (400–700 nm) and the UV spectrum (300–400 nm) to allow us to gain a better understanding of our UV paint treatment. Body condition for each individual was calculated as the residual from a regression of body weight versus length (*R*^2^ = 0.96, *t*_27_ = 24.20, *p* < 0.001). We detected no difference between the left and right meral spots of *N. oerstedii* in either spot hue or luminance (UV luminance: *χ*^2^ = 0.70, d.f. = 1, *p* = 0.40; visible luminance: *χ*^2^ = 0.22, d.f. = 1, *p* = 0.64; hue: *χ*^2^ = 1.56, d.f. = 1, *p* = 0.21), so four measurements (two from each arm) were averaged for each stomatopod for these analyses. In both luminance and hue analyses, we included sex, habitat, body condition and body length as fixed effects. Probability error distributions were Gaussian (identity link) for hue and gamma (log link) for luminance. A fully saturated model was fitted and the significance of each term in the model was assessed with Wald *χ*^2^-tests (R: Anova). Non-significant interaction terms were removed sequentially. Final model fit was assessed using deviance residual plots [35].

#### 2.4.2. Experimental manipulation

We also used GLMs for the experimental manipulation experiment to assess how UV reflectance of the meral spot (present/absent) and ability to detect chemical cues (present/absent) influenced intruder agonistic behaviours. For all these analyses, any pairs that did not interact were removed (final sample size: UV+/CC+, *n* = 6; UV+/CC−, *n* = 9; UV−/CC+, *n* = 8; UV−/CC−, *n* = 7). All models included UV (present/absent) and chemical cues (present/absent) as fixed effects. We did not observe any difference between male–male and female–female contests, so sex was not included as a factor. For each response variable, we assessed the significance of each term in the models with Wald *χ*^2^-tests and removed the interaction term if it was not significant. The probability error distributions fitted to the data were: gamma (log link) for latency until approach and speed of approach and binomial (logit link) for winner of fight. Duration of fight was analysed using a Weibull regression model and latency until first offensive behaviour using a linear model with the response variable log transformed. Duration in burrow was assessed using a linear model with fight duration as an offset. Proximity to burrow fitted a Poisson error distribution best; however, because it is not count data, we fitted both a Weibull distribution (next best fit) and a Poisson GLM with an observation level random effect to account for overdispersion (R: glmer). Both models produced similar results, but deviance residual plots suggested that the Poisson model fitted the data better. Thus, we report results from the Poisson model. Deviance residual plots were used to assess all model fits [35].

All count data were analysed with UV (present/absent) and chemical cues (present/absent) as fixed effects. To analyse offensive behaviour data, we used a GLM with a Poisson error distribution and an offset (duration of fight). Antennal flicks data fitted a Gaussian distribution best when it was converted to a rate and a log transformation was used. However, because it was count data, we also fitted a generalized linear mixed model (R: glmer) with a Poisson error distribution, an offset (time out of refuge during fight) and an observation level random effect account for over dispersion. Both models produced similar results; however, deviance residual plots suggested that the Gaussian model with a log transformation fitted the data better. Thus, we report results from Gaussian model. We assessed the significance of each term in the models with Wald *χ*^2^-tests and removed the interaction term if it was not significant. Defensive behavioural data were analysed using a zero inflated Poisson model (R: zeroinfl) with an offset (time out of refuge during fight). Likelihood ratio tests were used to assess whether the addition of each factor (UV or chemical cues) significantly improved the fit of the model compared to an intercept only model, and whether an interaction term improved the fit of the model compared to a main effects only model. Deviance residual plots were used to assess all model fits [35].

## 3. Results

### 3.1. Field study: variation in meral spot reflectance

The peak wavelength (hue) for *N. oerstedii* meral spots lies in the UV between 348 and 404 nm (minimum, maximum). We detected a weak trend towards longer stomatopods having meral spots with peaks at shorter wavelengths (*χ*^2^ = 3.68, d.f. = 1, *p* = 0.055; electronic supplementary material, figure S2*a*). Spot hue was not correlated with sex (*χ*^2^ = 0.04, d.f. = 1, *p* = 0.85), body condition (*χ*^2^ = 0.29, d.f. = 1, *p* = 0.59) or habitat (*χ*^2^ = 0.01, d.f. = 1, *p* = 0.91).

Both UV luminance and visible spectrum luminance of the meral spot differed between the sexes (figure 1*b*; UV: *χ*^2^ = 20.16, d.f. = 1, *p* < 0.001; visible: *χ*^2^ = 24.79, d.f. = 1, *p* < 0.001) as well as between the seagrass and rubble habitats (figure 1*b*; UV: *χ*^2^ = 12.08, d.f. = 1, *p* < 0.001; visible: *χ*^2^ = 23.46, d.f. = 1, *p* < 0.001). Furthermore, the relationship between habitat and UV spot luminance varied between the sexes (*χ*^2^ = 4.11, d.f. = 1, *p* = 0.043). Female spots were twice as bright in the visible spectrum compared with males, and stomatopods (both sexes) from rubble habitats had meral spots twice as bright in the visible spectrum compared with stomatopods from seagrass habitats. These main effects were also present in the UV portion of the spectrum, although with a smaller effect size than for the visible reflectance. UV reflectance was much greater for females from rubble habitats than for all other stomatopods. Body length had a weak, negative relationship with meral spot luminance in both visible (*χ*^2^ = 15.51, d.f. = 1, *p* < 0.001; electronic supplementary material, figure S2*b*) and UV wavelengths (*χ*^2^ = 9.40, d.f. = 1, *p* = 0.002; electronic supplementary material, figure S2*c*) while body condition did not correlate with UV or visible luminance (UV: *χ*^2^ = 0.01, d.f. = 1, *p* = 0.91; visible: *χ*^2^ = 0.17, d.f. = 1, *p* = 0.68).

### 3.2. Experimental manipulation: effect of UV reflectance and chemical cues on agonistic behaviours

As expected, manipulated meral spots (low UV reflectance) resulted in a much lower quantum catch for those photoreceptors with peak sensitivity in the UV, compared to control meral spots (figure 3*b*). Photoreceptors with peak sensitivity between 400 and 700 nm had a greater quantum catch for manipulated meral spots than for control meral spots (figure 3*b*). The luminance values (area under the spectral curve) for each treatment were 22.55 ± 2.4 for control and 28.69 ± 2.5 for manipulated (mean ± s.e.m.).

**Figure 4.**
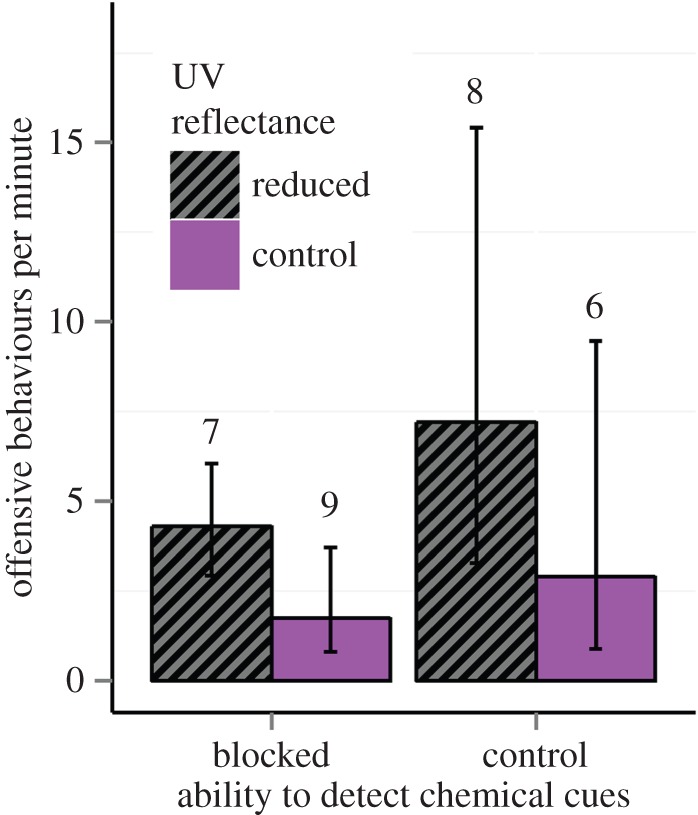
The effect of chemical cues and UV reflectance on the rate of offensive behaviours (mean, 95% CI) performed by *Neogonodactylus oerstedii* intruders towards residents. The intruder's ability to detect chemical cues was removed by dipping antennae in freshwater. The resident's meral spot UV reflectance was removed using a paint mix. Numbers indicate sample size.

**Figure 5.**
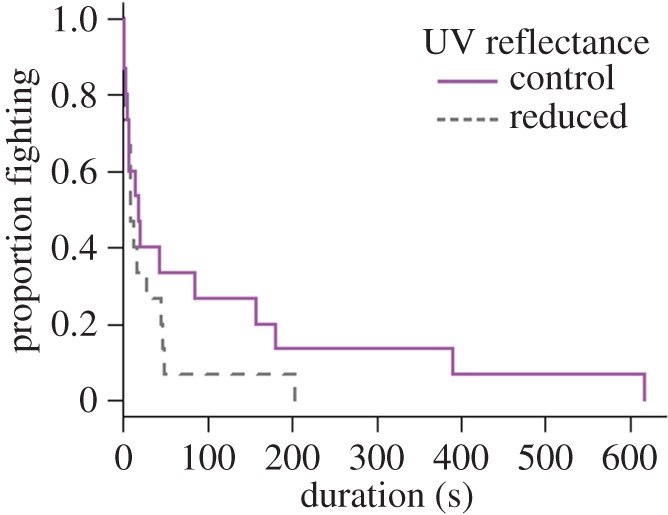
Proportion of *Neogonodactylus oerstedii* pairs still competing over a refuge after a certain amount of time. The resident's meral spot UV reflectance was removed using a paint mix (‘control’: *n* = 15; ‘reduced’: *n* = 15).

Altering UV reflectance changed the rate of the offensive behaviours performed by the intruder, with offensive behaviours performed more frequently when the UV reflectance of the resident's meral spot had been diminished (*χ*^2^ = 17.08, d.f. = 1, *p* < 0.001; figure 4). There was also a strong though not significant trend towards the resident's UV reflectance affecting fight duration (*χ*^2^ = 3.34, d.f. = 1, *p* = 0.067), with shorter fights occurring when UV reflectance had been removed (figure 5). This trend was mainly driven by a shorter latency until the intruder performed its first offensive behaviour when residents' UV reflectance had been removed (*F*_1,18_ = 4.99, *p* = 0.038). The presence or the absence of UV reflectance on the resident's meral spot did not affect any other behaviour measured, including the intruder's closest proximity to burrow, latency to approach, rate of defensive behaviours, antennal flicks, duration in burrow or the winner of the fight (table 1). Contests were generally won by residents, with residents winning 26 out of 31 contests.

**Table 1.** The effect of UV reflectance and chemical cues on agonistic behaviours of *N. oerstedii* intruders. Generalized linear models for each variable were assessed using Wald *χ*^2^-tests, except where noted. Each interaction was tested using a fully saturated model, and non-significant interaction terms were removed when testing main effects.

variable	model terms	*χ*^2^	d.f.	*p*-value
latency until approach	interaction	2.92	1	0.087
	UV	0.23	1	0.635
	chemical cues	<0.001	1	0.990
speed of approach	interaction	0.06	1	0.803
	UV	0.28	1	0.596
	chemical cues	4.61	1	0.032*
duration of fight	interaction	1.68	1	0.195
	UV	3.35	1	0.067
	chemical cues	0.01	1	0.928
offensive behaviours	interaction	0.96	1	0.326
	UV	17.08	1	<0.001*
	chemical cues	6.34	1	0.012*
defensive behaviours^a^	interaction	4.32	2	0.115
	UV	0.29	2	0.862
	chemical cues	2.13	2	0.345
antennal flicks	interaction	2.13	1	0.145
	UV	0.77	1	0.380
	chemical cues	0.07	1	0.784
proximity to burrow	interaction	1.96	1	0.161
	UV	0.49	1	0.482
	chemical cues	0.70	1	0.403
winner of fight	interaction	3.47	1	0.062
	UV	0.48	1	0.489
	chemical cues	2.07	1	0.150

*p < 0.05.

^a^Model terms were assessed using likelihood ratio tests.

**Figure 6.**
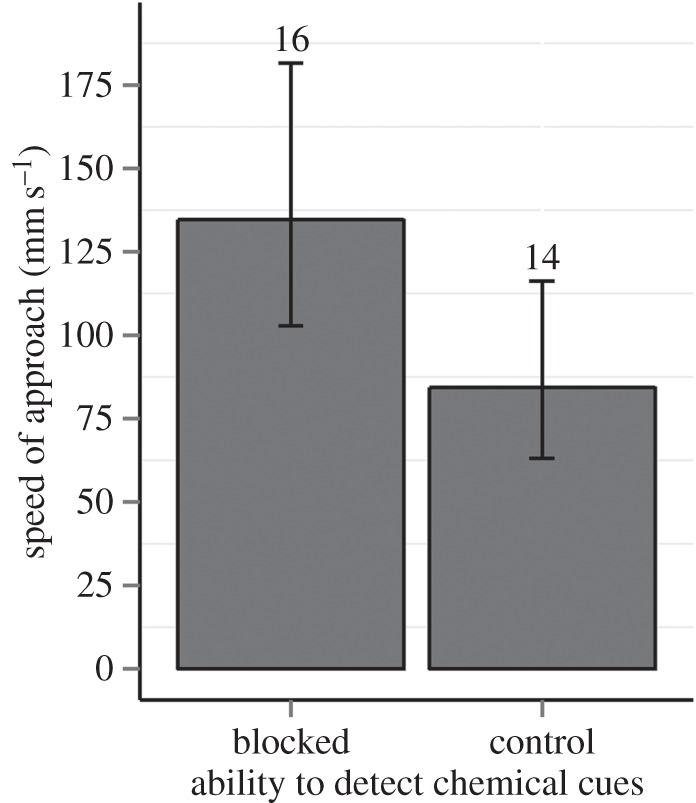
Speed of approach by intruder *Neogonodactylus oerstedii* towards a resident's refuge (mean, 95% CI) at the beginning of a fight. The intruder's ability to detect chemical cues was removed by dipping antennae in freshwater. Numbers indicate sample size.

When intruders' ability to detect chemical cues was removed, they approached the resident's refuge significantly more quickly (*χ*^2^ = 4.78, d.f. = 1, *p* = 0.029; figure 6). Stomatopods also performed fewer offensive behaviours in the absence of chemical cues (*χ*^2^ = 6.34, d.f. = 1, *p* = 0.012; figure 4). The presence or the absence of chemical cues did not affect any other variable measured, including proximity to burrow, latency to approach, duration of fight, defensive behaviours, duration in burrow or the winner of the fight (table 1). Surprisingly, there was no effect of availability of chemical cues on number of antennal flicks (table 1). There was also no significant interaction between UV reflectance and chemical cues for any behaviour recorded (table 1). Means and standard errors of all measured behaviours are provided in the electronic supplementary material, table S2.

## 4. Discussion

Complex signals have been demonstrated in several crustacean taxa including hermit crabs [36], *Aegla* decapods [37], fiddler crabs [38], blue crabs [6], crayfish [39], snapping shrimp [9] and copepods [40]. These signals are used across a variety of contexts, from predator/prey interactions [38] to mate choice [39], individual recognition [36] and agonistic interactions [9,37]. To the best of our knowledge, this is the first study to investigate complex signalling in stomatopods and to assess the signalling role of a specific chromatic signal in stomatopod communication.

We investigated whether the hue or luminance of stomatopods' meral spots could be used as a signal of sex, body condition or body length. Contrary to predictions, *N. oerstedii* spot hue, UV luminance and visible luminance correlated only weakly with stomatopod length and showed no correlation with body condition. Larger stomatopods had darker meral spots, with a trend towards a reflectance peak that was deeper in the UV. However, these weak correlations are unlikely to be used as a signal of body size by stomatopods. Stomatopods may have coarse colour vision, at least in the visible part of the spectrum. In laboratory feeding experiments, they only differentiated between colours at least 12–25 nm apart [19]. It is possible that in social contexts stomatopods perceive chromatic signals more accurately, and *N. oerstedii* does have five photoreceptor classes in the UV [17], potentially increasing colour discrimination across these wavelengths. However, lack of a strong correlation between hue and length suggests that hue would not be a reliable signal of body length. Luminance is also unlikely to be a useful signal of body size as it is also not strongly correlated to length. Furthermore, perceived luminance can vary dramatically depending on natural lighting or viewing conditions [41].

Female meral spots, however, are about twice as bright as male meral spots. Sexual dimorphism in meral spot colour has not previously been documented in stomatopods and it may allow *N. oerstedii* to use spot luminance as an indicator of sex. The efficacy of this signal might be further enhanced if the observed difference in spot luminance in different habitats increases contrast between the signal and the background coloration. It would be interesting to record rubble and seagrass reflectance to compare with the variations in meral spot reflectance. Conversely, such variation between habitats may make it difficult for a stomatopod to form a signal template for males and females. Instead the observed habitat difference in spot luminance may be due to prey availability. In crustaceans, many purple body colours are produced by crustacynanins [42]; like other carotenoids, these pigments must be acquired from prey items. If crustacyanin availability varies between habitats, this may explain the variation in meral spot luminance.

Despite luminance and hue being poor indicators of stomatopod body length and condition, the meral spot is clearly used as a signal in agonistic encounters. Experimental reduction of UV reflectance from residents' meral spots caused intruders (signal receivers) to perform a higher rate of offensive behaviours. This behavioural change may indicate that UV reflectance acts as a signal of aggressive intent or, alternatively, that it acts as an amplifier to enhance the aggressive signal contained in the meral spread display. If UV reflectance signals aggression levels, we would expect it to vary over short time scales. However, this is unlikely if this colour is produced by pigments or scattering structures in the exoskeleton, with reflectance probably fixed after each moult. Instead, we propose that UV reflectance is used to amplify the aggressive signal of the meral spread through one of two mechanisms. Colours are known to enhance displays in other species, particularly those that involve movement [43]. UV reflectance of the meral spot may be used to ensure the receiver detects the meral spread, a display known to signal aggressive intent [44]. Additionally, it may function to specifically draw attention to the raptorial appendages. Male jumping spiders use abdominal patterns which function to amplify differences in abdominal width [45]. While the patterns themselves do not indicate condition, they draw attention to the size of abdomen, which does vary with body condition. Similarly, the meral spot is located on the merus, a part of the raptorial appendage whose size may positively correlate with strike force [46]. Thus, drawing attention to this region might help the receiver to assess an opponent's fighting ability. Our results support the amplification hypothesis, because when UV reflectance was removed, intruder stomatopods increased their frequency of offensive behaviours.

We also observed a trend towards shorter fights when the resident's UV reflectance was removed. This was mainly due to a shorter latency before intruders performed their first offensive behaviour. Thus, stomatopod intruders appear more willing to engage in a fight when the resident's UV reflectance is low. This result further supports our suggestion that UV reflectance is used as a signal amplifier. In the absence of UV reflectance, stomatopods appear to underestimate aggression level and/or fighting ability and quickly escalated a contest. The increase in offensive behaviours throughout the remainder of the contest may help the intruder to reassess resident aggression.

Despite intruders subsequently performing a greater number of offensive behaviours during the fight, the winner of the refuge did not differ between treatment groups. This was as expected because we altered only the resident's signal, not its fighting ability. However, recent research in *N. bredini* has demonstrated that whichever stomatopod punches their opponent's telson more times (a behaviour known as *telson sparring*) is the stomatopod more likely to win the contest [47]. As we could not record resident behaviours inside the burrow, we were unable to quantify this behaviour. However, we did observe punches onto the opponent's telson, as described by Green & Patek [47]. It is possible that residents also increased the frequency of offensive behaviours in the UV reduction treatment group, in response to intruders performing more aggressive behaviours. Thus, contest resolution in *N. oerstedii* may also involve telson sparring, although further research is required to investigate the role of meral spot UV reflectance on telson sparring behaviour.

To investigate what components of the signal were altered, we conducted a visual analysis. This analysis identified two distinct changes in our meral spot manipulation that may be detectable by stomatopods: a reduction in UV reflectance and increased luminance in the visible spectrum. Current evidence suggests that stomatopods can perceive the change in UV reflectance [17,18], but it is unclear whether they can also perceive the increased luminance. Our manipulation resulted in an 85% reduction in UV reflectance. This is highly likely to be detected by stomatopods because our quantum catch analysis suggests three of the UV photoreceptor classes detected very little light, and the fourth detected less than half compared with controls (figure 3*b*). However, the 30% increase in visible spectrum luminance could also be perceived by stomatopods and may have contributed to the behavioural changes observed. While we know stomatopods can discriminate between wavelengths 12 and 25 nm apart in the visible range [19], we do not know how an increase in quantum catch in this range will be perceived. It has been suggested that luminance vision may be mediated by the photoreceptors in the dorsal and ventral regions of the eye [30]. These photoreceptors have broad sensitivity (350–600 nm [30]), but it is unknown how light information from these photoreceptors is processed or perceived. Without further knowledge about stomatopod luminance vision, we cannot determine if our attempt to standardize luminance was successful or if stomatopods could detect the increase in total luminance from the manipulation.

There were no other effects of UV reflectance reduction on intruder behaviour. Our limited sample size may have limited our ability to detect some effects. Nonetheless, behaviours recorded before the fight began, such as latency to approach and speed of approach, remained unchanged, suggesting that UV reflectance of the meral spot is not used to locate another stomatopod or detect the presence of another stomatopod in a refuge. In general, stomatopods perform the meral spread (which displays the meral spot) only during fights; therefore, we would not predict a difference in behaviours recorded before a fight began.

Our results are consistent with previous research on stomatopods demonstrating that chemical cues are used during agonistic encounters [21,22,48]. We found that intruders approached refuges faster when their ability to detect chemical cues was removed, suggesting that chemical cues are used to assess whether a potential refuge is currently occupied. Furthermore, the absence of chemical cues resulted in a lower rate of aggressive behaviours towards the resident. Stomatopods use chemical cues to assess opponent size [21] and for opponent recognition [22,49]. Many crustaceans use chemical cues to recognize conspecifics in a variety of contexts such as finding a mate [50,51], detecting opponents they have previously fought [22,52] or avoiding injured or diseased conspecifics [53]. If stomatopods use chemical cues to assess various aspects of opponent identity, they may be more hesitant to fight when these cues are absent. Similarly, using chemical cues to assess size would be advantageous because larger stomatopods have a stronger punch [46] and are more likely to win contests [21]. Because resident stomatopods remain partially concealed in the refuge, intruders may have difficulty assessing an opponent's size without the body size information encoded in chemical cues.

In this study, other intruder behaviours were unaffected by whether or not the intruder could detect chemical cues. Most surprising was the lack of difference in antennal flicks between treatment groups. Cheroske *et al*. [28] demonstrated that dipping antennae in freshwater causes a reduction in the number of antennal flicks in response to a stimulus (food or conspecific chemical cues). They conclude that freshwater treatment is an effective method of chemoreception ablation for up to 5 days [28]. The lack of difference in the number of antennal flicks between treatment groups in our study may be because many of the stomatopods entered the resident's cavity during the fight. During this time, we could not see the antennae or count any antennal flicks. It is likely that this influenced our ability to detect a difference between treatment groups. However, the other behavioural changes we observed between these treatment groups provide evidence that our treatment was effective.

These results suggest that chemical cues and visual cues comprise a multimodal signal in stomatopod agonistic encounters. We suggest that chemical cues are assessed first and are used to determine the presence of an opponent in a suitable refuge. During the fight, both chemical cues and meral spot reflectance are used. Intruders flick their antennae throughout the fight suggesting that they are continuously evaluating chemical cues, and both residents and intruders perform the meral spread indicating that continuous visual assessment also occurs. If chemical cues and meral spot reflectance were redundant, we would only expect to see an effect on stomatopod behaviour when both were absent. We did not observe this for any behavioural variable measured, suggesting that chemical cues and meral spot reflectance are not redundant. Rather, these results are consistent with the multiple messages hypothesis, in which each signal encodes a different message [1]. Our results indicate that in stomatopod agonistic encounters, meral spot UV reflectance and/or luminance are used to amplify the meral spread threat display, whereas chemical cues indicate size [21] and opponent identity [22,49]. Using different modalities to send multiple messages can be beneficial to both signaller and receiver by ensuring the receiver responds appropriately. In this case, the intruder (receiver) can modulate its own aggressive response using both UV reflectance and chemical cues; the UV reflectance enhances the meral spread display, allowing the stomatopod to quickly assess the opponent's aggressive state while chemical cues allow the intruder to assess fighting ability and opponent identity.

## Supplementary Material

Supplemental Material: supplementary tables and figures

## Data accessibility

Data can be accessed on Dryad: http://dx.doi.org/10.5061/dryad.53c84.

## References

[1] Hebets EA, Papaj DR 2005 Complex signal function: developing a framework of testable hypotheses. Behav. Ecol. Sociobiol. 57, 197–214. (doi:10.1007/s00265-004-0865-7)

[2] McGraw KJ, Hill GE 2000 Differential effects of endoparasitism on the expression of carotenoid- and melanin-based ornamental coloration. Proc. R. Soc. Lond. B 267, 1525–1531. (doi:10.1098/rspb.2000.1174) 10.1098/rspb.2000.1174 PMC1690705 11007328

[3] Moller AP, Pomiankowski A 1993 Why have birds got multiple sexual ornaments? Behav. Ecol. Sociobiol. 32, 167–176. (doi:10.1007/BF00173774)

[4] Caldwell MS, Johnston GR, McDaniel JG, Warkentin KM 2010 Vibrational signaling in the agonistic interactions of red-eyed treefrogs. Curr. Biol. 20, 1012–1017. (doi:10.1016/j.cub.2010.03.069) 20493702 10.1016/j.cub.2010.03.069

[5] Scheuber H, Jacot A, Brinkhof MWG 2004 Female preference for multiple condition-dependent components of a sexually selected signal. Proc. R. Soc. Lond. B 271, 2453–2457. (doi:10.1098/rspb.2004.2907) 10.1098/rspb.2004.2907 PMC1691884 15590595

[6] Baldwin J, Johnsen S 2012 The male blue crab, *Callinectes sapidus*, uses both chromatic and achromatic cues during mate choice. J. Exp. Biol. 215, 1184–1191. (doi:10.1242/jeb.067512) 22399664 10.1242/jeb.067512

[7] Rypstra AL, Schlosser AM, Sutton PL, Persons MH 2009 Multimodal signalling: the relative importance of chemical and visual cues from females to the behaviour of male wolf spiders (Lycosidae). Anim. Behav. 77, 937–947. (doi:10.1016/j.anbehav.2008.12.026)

[8] Ballentine B, Searcy WA, Nowicki S 2008 Reliable aggressive signalling in swamp sparrows. Anim. Behav. 75, 693–703. (doi:10.1016/j.anbehav.2007.07.025)

[9] Hughes M 1996 The function of concurrent signals: visual and chemical communication in snapping shrimp. Anim. Behav. 52, 247–257. (doi:10.1006/anbe.1996.0170)

[10] Preininger D, Boeckle M, Freudmann A, Starnberger I, Sztatecsny M, Hödl W 2013 Multimodal signaling in the small torrent frog (*Micrixalus saxicola*) in a complex acoustic environment. Behav. Ecol. Sociobiol. 67, 1449–1456. (doi:10.1007/s00265-013-1489-6) 23956486 10.1007/s00265-013-1489-6 PMC3742427

[11] Rowe C 2002 Sound improves visual discrimination learning in avian predators. Proc. R. Soc. Lond. B 269, 1353–1357. (doi:10.1098/rspb.2002.2012) 10.1098/rspb.2002.2012 PMC1691037 12079658

[12] Roberts JA, Taylor PW, Uetz GW 2007 Consequences of complex signaling: predator detection of multimodal cues. Behav. Ecol. 18, 236–240. (doi:10.1093/beheco/arl079)

[13] Bro-Jørgensen J, Dabelsteen T 2008 Knee-clicks and visual traits indicate fighting ability in eland antelopes: multiple messages and back-up signals. BMC Biol. 6, 47 (doi:10.1186/1741-7007-6-47) 18986518 10.1186/1741-7007-6-47 PMC2596769

[14] Elwood RW, Briffa M 2001 Information gathering and communication during agonistic encounters: a case study of hermit crabs. Adv. Stud. Behav. 30, 53–97. (doi:10.1016/S0065-3454(01)80005-X)

[15] Bok M 2014 The physiological, ecological and evolutionary basis of polychromatic ultraviolet sensitivity in stomatopod crustaceans (Doctoral dissertation). Retrieved from ProQuest database (UMI No. 3609836).

[16] Marshall J, Arikawa K 2014 Unconventional colour vision. Curr. Biol. 24, 1150–1154. (doi:10.1016/j.cub.2014.10.025) 10.1016/j.cub.2014.10.025 25514002

[17] Bok MJ, Porter ML, Place AR, Cronin TW 2014 Biological sunscreens tune polychromatic ultraviolet vision in mantis shrimp. Curr. Biol. 24, 1636–1642. (doi:10.1016/j.cub.2014.05.071) 24998530 10.1016/j.cub.2014.05.071

[18] Marshall NJ, Oberwinkler J 1999 Ultraviolet vision: the colourful world of the mantis shrimp. Nature 401, 873–874. (doi:10.1038/44751) 10553902 10.1038/44751

[19] Thoen HH, How MJ, Chiou T-H, Marshall J 2014 A different form of color vision in mantis shrimp. Science 343, 411–413. (doi:10.1126/science.1245824) 24458639 10.1126/science.1245824

[20] Chiou T-H, Marshall NJ, Caldwell RL, Cronin TW 2011 Changes in light-reflecting properties of signalling appendages alter mate choice behaviour in a stomatopod crustacean *Haptosquilla trispinosa*. Mar. Freshwater Behav. Phys. 44, 1–11. (doi:10.1080/10236244.2010.546064)

[21] Caldwell RL 1987 Assessment strategies in stomatopods. Bull. Mar. Sci. 41, 135–150.

[22] Caldwell RL 1985 A test of individual recognition in the stomatopod *Gonodactylus festae*. Anim. Behav. 33, 101–106. (doi:10.1016/s0003-3472(85)80123-8)

[23] Caldwell RL 1979 Cavity occupation and defensive behaviour in the stomatopod *Gonodactylus festae*: evidence for chemically mediated individual recognition. Anim. Behav. 27, 194–201. (doi:10.1016/0003-3472(79)90139-8)

[24] Hansen HJ 1895 Isopoden, Cumaceen u. Stomatopoden der plankton-expedition. Berlin, Germany: Deutsche Akademie der Wissenschaften.

[25] Tedetti M, Sempéré R 2006 Penetration of ultraviolet radiation in the marine environment. A review. Photochem. Photobiol. 82, 389–397. (doi:10.1562/2005-11-09-IR-733) 16613490 10.1562/2005-11-09-IR-733

[26] Endler JA 1990 On the measurement and classification of colour in studies of animal colour patterns. Biol. J. Linn. Soc. 41, 315–352. (doi:10.1111/j.1095-8312.1990.tb00839.x)

[27] Duntley SQ 1960 The visibility of submerged objects. La Jolla, CA: Scripps Institution of Oceanography Technical Report.

[28] Cheroske AG, Cronin TW, Durham MF, Caldwell RL 2009 Adaptive signaling behavior in stomatopods under varying light conditions. Mar. Freshwater Behav. Phy. 42, 219–232. (doi:10.1080/10236240903169222)

[29] Vorobyev M, Marshall J, Osorio D, de Ibarra NH, Menzel R 2001 Colourful objects through animal eyes. Color Res. Appl. 26, S214–S217. (doi:10.1002/1520-6378(2001)26:1+<::AID-COL45>3.0.CO;2-A)

[30] Marshall J, Cronin TW, Kleinlogel S 2007 Stomatopod eye structure and function: a review. Arthropod Struct. Dev. 36, 420–448. (doi:10.1016/j.asd.2007.01.006) 18089120 10.1016/j.asd.2007.01.006

[31] Dingle H, Caldwell RL 1969 The aggressive and territorial behaviour of the mantis shrimp *Gonodactylus bredini* Manning (Crustacea: Stomatopoda). Behaviour 33, 115–136. (doi:10.2307/4533261) 5815890 10.1163/156853969x00341

[32] R Core Team. 2014 R: a language and environment for statistical computing. Vienna, Austria: R Foundation for Statistical Computing.

[33] Montgomerie R 2008 CLR v. 1.05. Queens University, Kingston, Canada.

[34] Montgomerie R 2008 RCLR, v. 0.9.28. Queen's University, Kingston, Canada.

[35] Zuur AF 2009 Mixed effects models and extensions in ecology with R. New York, NY: Springer.

[36] Gherardi F, Tiedemann J 2004 Chemical cues and binary individual recognition in the hermit crab *Pagurus longicarpus*. J. Zool. 263, 23–29. (doi:10.1017/S0952836904004807)

[37] Palaoro AV, Ayres-Peres L, Santos S 2013 Modulation of male aggressiveness through different communication pathways. Behav. Ecol. Sociobiol. 67, 283–292. (doi:10.1007/s00265-012-1448-7)

[38] Chiussi R, Diaz H 2002 Orientation of the fiddler crab, *Uca cumulanta*: responses to chemical and visual cues. J. Chem. Ecol. 28, 1787–1796. (doi:10.1023/A:1020561101616) 12449506 10.1023/a:1020561101616

[39] Aquiloni L, Gherardi F 2008 Assessing mate size in the red swamp crayfish *Procambarus clarkii*: effects of visual versus chemical stimuli. Freshwater Biol. 53, 461–469. (doi:10.1111/j.1365-2427.2007.01911.x)

[40] Yen J, Weissburg MJ, Doall MH 1998 The fluid physics of signal perception by mate-tracking copepods. Phil. Trans. R. Soc. Lond. B 353, 787–804. (doi:10.1098/rstb.1998.0243) 9652126 10.1098/rstb.1998.0243 PMC1692257

[41] Endler JA 1993 The color of light in forests and its implications. Ecol. Monogr. 63, 2–27. (doi:10.2307/2937121)

[42] Bandaranayake WM 2006 The nature and role of pigments of marine invertebrates. Nat. Prod. Rep. 23, 223–255. (doi:10.1039/b307612c) 16572229 10.1039/b307612c

[43] Fleishman LJ 2000 Signal function, signal efficiency and the evolution of anoline lizard dewlap color. In Animal signals: signalling and signal design in animal communication, pp. 209–236. Trondheim, Norway: Tapir Academic.

[44] Dingle H 1969 A statistical and informational analysis of aggressive communication in the mantis shrimp *Gonodactylus bredini* Manning. Anim. Behav. 17, 561–575. (doi:10.1016/0003-3472(69)90165-1) 10.1163/156853969x00341 5815890

[45] Taylor PW, Hasson O, Clark DL 2000 Body postures and patterns as amplifiers of physical condition. Proc. R. Soc. Lond. B 267, 917–922. (doi:10.1098/rspb.2000.1090) 10.1098/rspb.2000.1090 PMC1690626 10853735

[46] Claverie T, Chan E, Patek SN 2011 Modularity and scaling in fast movements: power amplification in mantis shrimp. Evolution 65, 443–461. (doi:10.1111/j.1558-5646.2010.01133.x) 20840593 10.1111/j.1558-5646.2010.01133.x

[47] Green PA, Patek SN 2015 Contests with deadly weapons: telson sparring in mantis shrimp (Stomatopoda). Biol. Lett. 11, 20150558 (doi:10.1098/rsbl.2015.0558) 26399976 10.1098/rsbl.2015.0558 PMC4614432

[48] Caldwell RL 1982 Interspecific chemically mediated recognition in two competing stomatopods. Mar. Behav. Physiol. 8, 189–197. (doi:10.1080/10236248209387016)

[49] Caldwell RL 1992 Recognition, signalling and reduced aggression between former mates in a stomatopod. Anim. Behav. 44, 11–19. (doi:10.1016/s0003-3472(05)80749-3)

[50] Ting JH, Snell TW 2003 Purification and sequencing of a mate-recognition protein from the copepod *Tigriopus japonicus*. Mar. Biol. 143, 1–8. (doi:10.1007/s00227-003-1071-2)

[51] Gleeson RA 1991 Intrinsic factors mediating pheromone communication in the blue crab, *Callinectes sapidus*. In Crustacean sexual biology, pp. 17–32. New York, NY: Columbia University Press.

[52] Johnson ME, Atema J 2005 The olfactory pathway for individual recognition in the American lobster *Homarus americanus*. J. Exp. Biol. 208, 2865–2872. (doi:10.1242/jeb.01707) 16043591 10.1242/jeb.01707

[53] Behringer DC, Butler MJ, Shields JD 2006 Ecology: avoidance of disease by social lobsters. Nature 441, 421 (doi:10.1038/441421a) 16724051 10.1038/441421a

