# Supplementary material for: Multimodal signals: ultraviolet reflectance and chemical cues in stomatopod agonistic encounters
Source: R Soc Open Sci. 2016 Aug 3;3(8):160329. doi: 10.1098/rsos.160329 (PMC5108963; doi:10.1098/rsos.160329)
Supplement: Supplemental Material: supplementary tables and figures [file rsos160329supp1.docx]

**Multimodal signals: Ultraviolet reflectance and chemical cues in stomatopod agonistic encounters**

*Amanda M. Franklin, N. Justin Marshall & Sara M. Lewis*

**Supplemental Information**

**Table S1: Statistical models used to assess the effect of various factors on meral spot colour and agonistic behaviours.** Sample size is indicated for different categorical treatment groups. Saturated models (including all interactions) were assessed first and non-significant interaction terms were removed. Final models used to analyse data are indicated.

| **Experiment** | **Variables** | **Sample Size** | **Final Model** |
| --- | --- | --- | --- |
| *Field Study*:  Reflectance spectra of meral spot | Sex,  Habitat,  Length,  Body condition | Female, rubble: 10  Female, seagrass: 6  Male, rubble: 4  Male, seagrass: 9 | *Hue and visible luminance:*  ~ Sex + Habitat + Length + Body condition  *UV luminance:*  ~ Sex + Habitat + Length + Body condition |
| *Manipulation*:  Effect of UV reflectance and chemical cues on agonistic behaviours | UV treatment,  Chemical cue treatment | UV+, CC+: 6  UV+, CC-: 9  UV-, CC+: 8  UV-, CC-: 7 | *All variables (see Table S2):*  ~ UV + CC |

**Table S2: Means and SEM for all behaviours measured in each of the four treatment groups.** UV+: UV reflectance of the meral spot is present; UV-: UV reflectance of the meral spot has been removed; CC+: the intruder can detect chemical cues; CC-: the intruder cannot detect chemical cues.

|  | **Treatment** | | | |
| --- | --- | --- | --- | --- |
| **Behaviour** | **UV+, CC+**  *(Control)* | **UV+, CC-**  *(Effect of chemical cues)* | **UV-, CC+**  *(Effect of UV)* | **UV- , CC-**  *(Effect of UV & chemical cues)* |
| Latency until approach (s) | 157.17 ± 57.30 | 300.44 ± 111.68 | 249.25 ± 84.89 | 138.57 ± 39.29 |
| Speed of approach (mm/s) | 92.38 ± 32.87 | 138.28 ± 25.01 | 78.08 ± 10.67 | 129.91 ± 24.27 |
| Duration of fight (s) | 147.00 ± 97.41 | 74.56 ± 43.64 | 18.00 ± 6.89 | 43.43 ± 27.35 |
| Latency until offensive (s) | 12.67 ± 6.72 | 6.20 ± 2.13 | 1.00 ± 0.50 | 3.17 ± 2.40 |
| Offensive behaviours (#/min): |  |  |  |  |
| - *Punch* | 0.50 ± 0.25 | 0.16 ± 0.12 | 0.00 ± 0.00 | 0.08 ± 0.08 |
| - *Lunge* | 6.26 ± 4.82 | 3.02 ± 2.84 | 0.85 ± 0.51 | 1.93 ± 1.69 |
| - *Meral spread* | 16.92 ± 9.84 | 4.89 ± 3.25 | 3.77 ± 2.15 | 8.80 ± 3.92 |
| - *Punch and lunge* | 1.01 ± 0.89 | 1.69 ± 1.13 | 0.00 ± 0.00 | 2.23 ± 1.66 |
| Curls (#/min) | 5.76 ± 4.87 | 4.01 ± 2.23 | 3.51 ± 2.42 | 4.82 ± 4.23 |
| Antennal flicks (#/min) | 23.68 ± 12.90 | 14.93 ± 10.04 | 4.64 ± 3.74 | 15.85 ± 6.08 |
| Closest proximity to burrow (mm) | 8.87 ± 8.87 | 16.69 ± 16.40 | 68.74 ± 51.83 | 3.74 ± 3.74 |
| Proportion of fights intruder won | 0.17 ± 0.17 | 0.11 ± 0.11 | 0.00 ± 0.00 | 0.43 ± 0.20 |


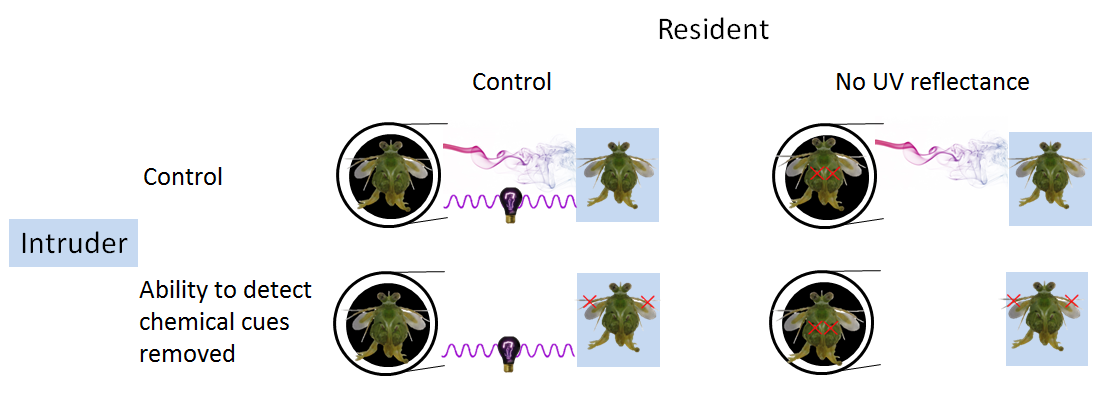


**Figure S1: Schematic of experimental design shams of the four treatment groups.** In each pair, resident is on left in an artificial refuge and intruder is on the right. Top left: control (UV and chemical cues present); Top right: effect of UV reflectance (resident UV reflectance diminished using sunscreen, chemical cues present); Bottom left: effect of chemical cues (UV reflectance present, intruder’s ability to detect chemical cues removed using freshwater); Bottom right: combined effect of UV reflectance and chemical cues (both UV reflectance and chemical cues removed).


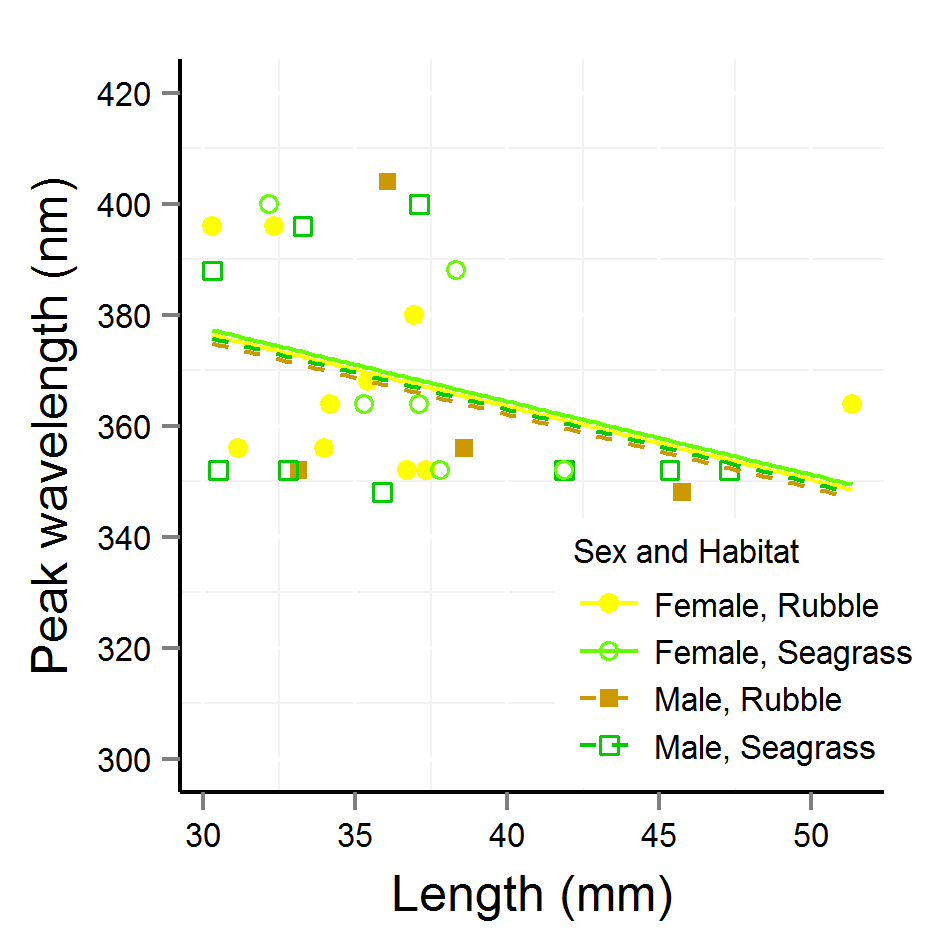


**C**

**B**

**A**


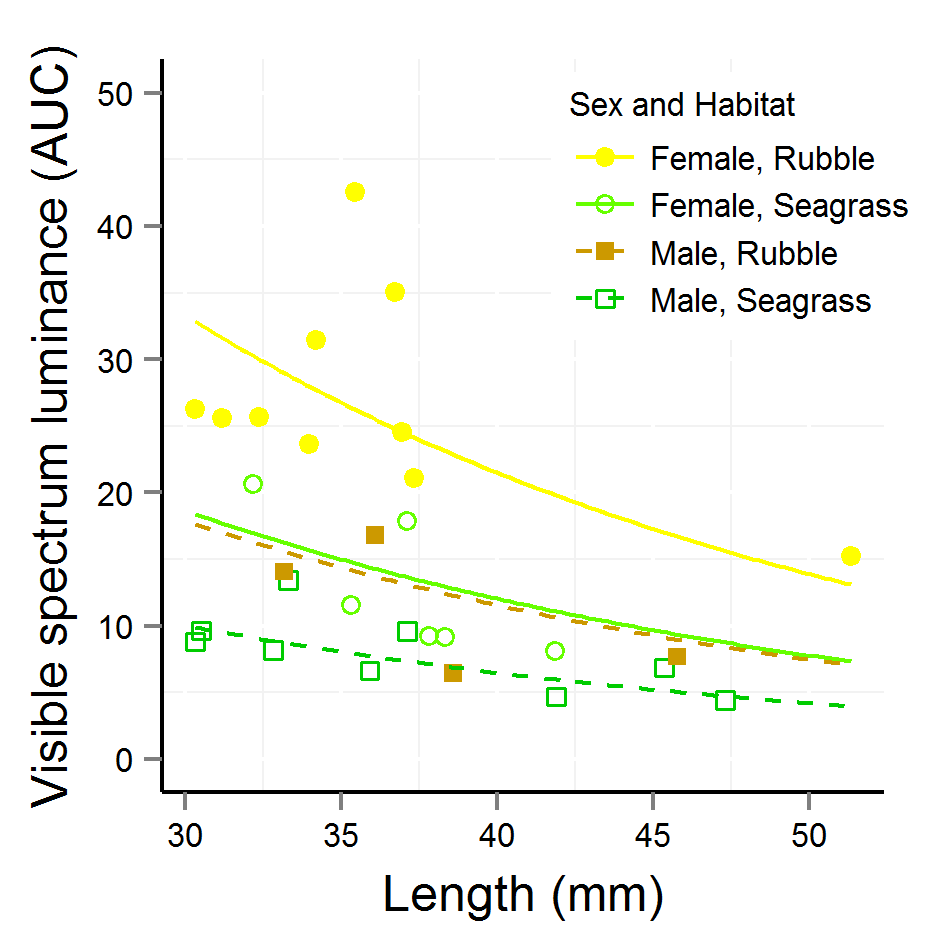

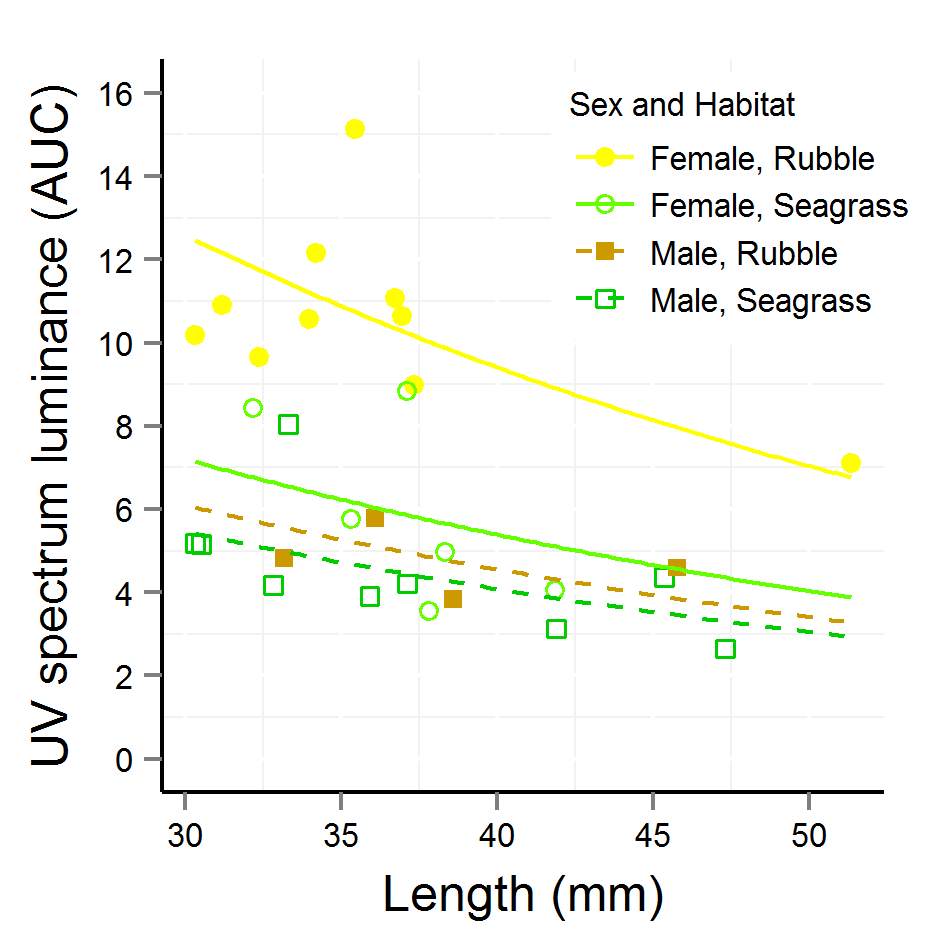


**Figure S2: Correlations of length with meral spot (A) hue (peak wavelength), (B) visual spectrum luminance and (C) UV spectrum luminance.** Line indicates the prediction from the statistical model for females (circles and solid lines) and males (squares and dashed lines) collected from seagrass (green and open points) and rubble (yellow and closed points). Luminance calculated as area under the spectral curve between 300 – 400 nm for UV and 400 – 700 nm for visible.
